# Implementation and evaluation of the centering pregnancy group prenatal care model in pregnant women with diabetes: a convergent parallel mixed methods study protocol

**DOI:** 10.1186/s12978-024-01792-3

**Published:** 2024-04-18

**Authors:** Mahsa Maghalian, Fatemeh Abbasalizadeh, Sakineh Mohammad-Alizadeh-Charandabi, Solmaz Ghanbari-Homaie, Mojgan Mirghafourvand

**Affiliations:** 1 Department of Midwifery, Faculty of Nursing and Midwifery, Tabriz University of Medical Sciences, Tabriz, Iran; 2 Women’s Reproductive Health Research Center, Department of Perinatology, Faculty of Medicine, Tabriz University of Medical Sciences, Tabriz, Iran; 3 Social Determinants of Health Research Center, Faculty of Nursing and Midwifery, Tabriz University of Medical Sciences, Tabriz, Iran

**Keywords:** Antenatal care, Group care, Diabetes, Pregnancy

## Abstract

**Background:**

Diabetes during pregnancy has negative effects on both mothers and their fetuses. To improve perinatal outcomes and women’s experience of care, the World Health Organization (WHO) suggests implementing health system interventions to enhance the use and quality of antenatal care. The main goal of this study is to implement and evaluate the outcomes of the Centering Pregnancy group care model for pregnant women with diabetes.

**Methods/design:**

The study will consist of three phases: a quantitative phase, a qualitative phase, and a mixed phase. In the quantitative phase, a randomized controlled trial will be conducted on 100 pregnant women with diabetes receiving prenatal care in Tabriz City, Iran. The Summary of Diabetes Self-Care Activities (SDSCA) questionnaire will also be validated in this phase. The qualitative phase will use qualitative content analysis with in-depth and semi-structured individual interviews to explore pregnant women’s understanding of the impact of the Centering Pregnancy group care model on their care process. The mixed phase will focus on the degree and extent of convergence between quantitative and qualitative data.

**Discussion:**

The implementation of the Centering Pregnancy group care approach is anticipated to empower women in effectively managing their diabetes during pregnancy, resulting in improved outcomes for both mothers and newborns. Furthermore, adopting this approach has the potential to alleviate the financial burden of diabetes on healthcare system.

**Trial registration:**

Iranian Registry of Clinical Trials (IRCT): (IRCT20120718010324N80/ Date of registration: 2024-01-03). URL: https://irct.behdasht.gov.ir/trial/74206.

## Plain Language Summary

Diabetes during pregnancy, whether pre-existing or gestational, can lead to complications for both the mother and the baby. Gestational diabetes is common and poses risks such as preterm birth and cesarean delivery. Pre-existing diabetes is on the rise globally and increases the likelihood of adverse outcomes like fetal death and birth defects. Centering Pregnancy is a group-based prenatal care model that offers comprehensive care to women with similar gestational ages. It promotes behavior modification, social support, and knowledge exchange among participants. However, there is limited research on the effectiveness of this model specifically for women with diabetes, especially in Iran.

To address this research gap, the study aims to implement and evaluate the Centering Pregnancy model in pregnant women with diabetes in Iran. By employing a combined methodology, the researchers will assess the current state of care and gather comprehensive data to understand the impact of the model. The findings of this study can contribute to improving the healthcare system’s burden and enhancing self-care practices for pregnant women with diabetes, ultimately leading to better pregnancy experiences and improved maternal and neonatal outcomes.

## Background

Pregnancy involves multiple changes in the structure and function of the mother’s body, serving as a biological stress test for her various systems and organs [1]. Diabetes during pregnancy can be categorized into two subgroups: pre-existing diabetes, which includes type 1 or type 2 diabetes, and gestational diabetes mellitus, which is diabetes that develops during pregnancy [2]. Both gestational and pre-existing diabetes result in hyperglycemia, with pre-existing diabetes posing a greater severity and detrimental effects on the health of both mother and fetus [3].

Gestational diabetes is a common complication during pregnancy, typically occurring after the first trimester [4]. It affects around 14% of women globally and has a prevalence of 7.6% in Iran [5, 6]. Gestational diabetes raises the likelihood of negative consequences for both the mother and the baby, including risks of macrosomia, preterm birth, fetal loss, and cesarean delivery. Additionally, it can lead to complications like shoulder dystocia, birth trauma, and neonatal hypoglycemia. Women with gestational diabetes also face a 50% lifetime risk of developing type 2 diabetes [7, 8]. The prevalence of pre-existing diabetes in pregnancy is rising globally, despite being relatively uncommon [9]. While effective blood sugar control during pregnancy can reduce the risk of adverse perinatal outcomes and birth defects, women with pre-existing diabetes remain at a higher risk for unfavorable pregnancy outcomes, including fetal death, maternal preeclampsia, and birth defects [10, 11].

Improving maternal health and reducing child mortality are among the key goals of the United Nations’ Millennium Development Goals [12]. Group-based prenatal care, such as Centering Pregnancy, is a model that leads to a reduction in preterm labor and adverse birth outcomes among women with uncomplicated pregnancies [13]. Moreover, pregnant women with common medical conditions like diabetes who participate in group prenatal care are not at a higher risk of preterm birth, low birth weight, or neonatal intensive care unit (NICU) hospitalization [14].

Centering Pregnancy is an innovative prenatal care model that involves grouping women with similar gestational ages for comprehensive care. This approach fosters behavior modification, social support, and the exchange of knowledge among participants. By creating a positive environment, pregnant women can learn from one another, gain a greater sense of agency over their pregnancies, and benefit from improved education and support. Centering Pregnancy stands out as a distinct and advantageous approach to providing prenatal care [15–17].

Currently, there is a research gap in evaluating the Centering Pregnancy group care model specifically for diabetes due to limited studies conducted in this area [18, 19]. Most of the existing studies have relied on observational designs [20–22], which may be susceptible to confounding factors. Moreover, the majority of studies have been conducted in other countries, particularly in the United States, and findings based on different racial and ethnic groups may not be directly applicable to Iranian women. However, it has been demonstrated that this model can reduce neonatal hospitalization costs by decreasing NICU admissions [23].

Therefore, given the adverse consequences of diabetes in pregnancy, implementing the Centering Pregnancy group care model has the potential to be beneficial in improving the healthcare system’s burden. To enhance self-care practices for pregnant women with diabetes and improve their pregnancy experience, as well as maternal and neonatal outcomes, it is essential to assess the current state of care and gather insights from these individuals using a comprehensive approach. This study will serve as the first investigation in Iran to implement and evaluate the Centering Pregnancy group care model in pregnant women with diabetes, employing a combined methodology to gather comprehensive data.

## Objectives

The overall objective of this study is to implement and evaluate the outcomes of the Centering Pregnancy group care model in pregnant women with diabetes.

## Study design

**Fig. 1:**
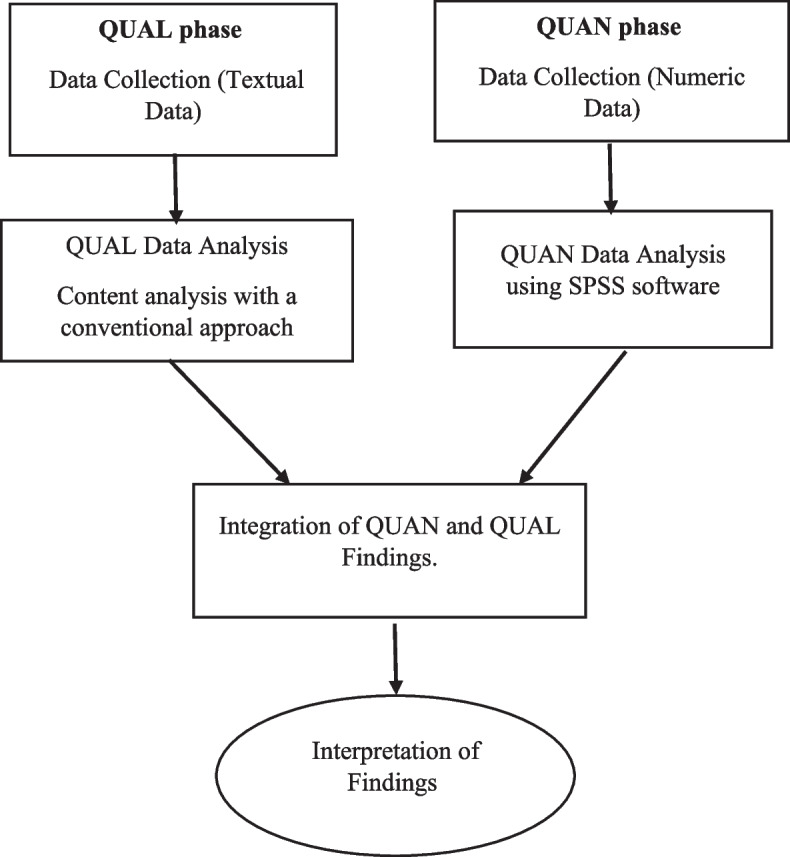
The mixed methods convergent parallel design

This research is a study of a convergent parallel mixed methods, and its paradigm is pragmatism. The present study will be conducted in three phases: quantitative, qualitative, and the combination of both quantitative and qualitative phases. In this mixed design, qualitative and QUAN quantitative data will be collected and analyzed simultaneously and independently. Both types of data will have equal priority and value (QUAL + QUAN). Data analysis will be performed separately, and the results will be integrated during the data interpretation stage. The discussion will focus on the degree and extent of convergence between quantitative and qualitative data [24, 25] (Fig. 1).

## Study phases

### Quantitative phase (phase 1)

#### Study design and setting

**Fig. 2:**
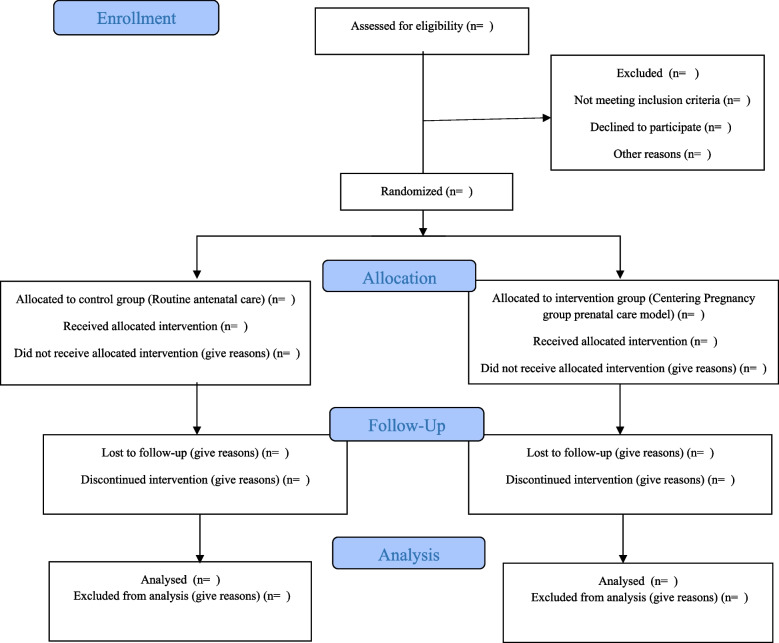
Consort flowchart of the trial process

The quantitative phase of the present study will involve a randomized controlled trial conducted on 100 pregnant women with diabetes who seek prenatal care at educational and therapeutic hospitals such as Taleghani, Al-Zahra, 29 Bahman, Al-Ghadir (governmental hospitals), as well as Valiasr and Behbood (private hospitals), and health centers in Tabriz city-Iran. Figure 2 shows the flowchart of this phase.

#### Specific objectives

The specific objectives of the quantitative phase are as follows:

1. To compare the mean scores of diabetes self-care activities in weeks 36–39 of pregnancy between the intervention and control groups (receiving Centering Pregnancy group care model and receiving routine care, respectively), while controlling for baseline scores.
2. To compare the mean scores of self-efficacy in weeks 36–39 of pregnancy between the intervention and control groups (receiving Centering Pregnancy group care model and receiving routine care, respectively), while controlling for baseline scores.
3. To compare the mean scores of pregnancy experience in weeks 36–39 of pregnancy between the intervention and control groups (receiving Centering Pregnancy group care model and receiving routine care, respectively), while controlling for baseline scores.

#### Secondary objectives

The secondary objectives of the quantitative phase are as follows:

1. To compare the frequency of preterm deliveries between the intervention and control groups.
2. To compare the frequency of cesarean deliveries between the intervention and control groups.
3. To compare the frequency of admission in the NICU between the intervention and control groups.
4. To compare the mean satisfaction scores regarding perinatal care between the intervention and control groups.
5. To compare the mean scores of breastfeeding performance at 6 weeks postpartum between the intervention and control groups.
6. To determine the face validity, content validity, construct validity, and reliability of the Summary of the Diabetes Self-Care Activities (SDSCA) questionnaire in women with gestational diabetes.

#### Eligibility criteria

The study includes pregnant women who have gestational diabetes mellitus (GDM) or type 2 diabetes and are at least 24 weeks pregnant. They must be able to attend group sessions in Tabriz city for a duration of 8 weeks before childbirth. However, the study excludes women with multiple pregnancies, major fetal anomalies, concurrent medical conditions, psychiatric disorders, and advanced or complicated diabetes. These criteria are important for ensuring the study focuses on a specific group of participants who meet the requirements and can provide valuable insights into the research objectives.

#### Sample size

The sample size in this study was calculated based on the variable of diabetes self-care activities using the G-Power software. According to the results of the study by Al-Hashmi et al. [26] regarding the variable of diabetes self-care activities, and considering M_1_ = 3.1 and M_2_ = 3.7 (assuming a 25% increase due to intervention), SD_1_ = 1.2 and SD_2_ = 1.2, two-sided α = 0.05, and Power = 90%, the sample size was calculated to be 45 participants. Taking into account a 10% dropout rate, a final sample size of 50 participants in each group was considered.

#### Procedure

A comprehensive description of the research, including its objectives and the methods of implementation, will be provided to the participants. Detailed explanations regarding the benefits of implementing this model in improving lifestyle and mitigating the consequences of diabetes on both the mother and the fetus will be emphasized. Moreover, it will be emphasized that no adverse effects have been observed on women and their fetuses as a result of this study. Additionally, the costs associated with transportation for attending sessions will be covered to encourage active participation. Those interested in participating will be asked to provide informed written consent through a consent form. Strict confidentiality and anonymity will be maintained in reporting the results, ensuring the privacy of participants. Participants will also be informed of their right to withdraw from the study at any time without facing any penalties or obligations. Subsequently, for those willing to participate, they will be assigned to the study groups accordingly.

#### Randomization and blinding

To allocate participants to the study groups, a stratified block randomization method will be employed, based on the classification of gestational diabetes and type 2 diabetes. The block sizes will be either 4 or 6, and the allocation ratio will be 1:1. To ensure concealment of allocation, a central allocation method will be used. Specifically, certain participant information will be sent via text message to the supervisor, who will then communicate the participant’s group assignment. Regarding blinding, the study will be conducted as a single-blind study. Due to the nature of the intervention, participants, clinical caregivers, outcome assessors, and intervention providers will not be blinded to group allocation. However, efforts will be made to minimize bias during data analysis by blinding the data analyst to group allocation. This will be achieved through participant coding, preventing the analyst from accessing information about which participants belong to each group. By doing so, the analysis and interpretation of the data will remain impartial and unbiased.

#### Intervention

The intervention group will participate in an 8-week program consisting of group sessions held every 2 weeks, with each session lasting approximately two hours. These sessions will be led by a midwife who is a PhD student in midwifery and an obstetrician, and the group will consist of 2 to 10 women. The content of each session will be based on the curriculum and materials of the centering pregnancy program, which have been reviewed and approved by the obstetrician and nutritionist.

At the start of each session, the midwife, assisted by the researcher, will conduct measurements of weight, blood pressure, and blood glucose levels using a glucometer. This assessment process will take approximately 30 min. The remaining time, around 60 to 90 min, will be dedicated to group discussions focused on predetermined topics that are relevant to the participants’ needs.

- Session 1: The first session will provide diabetes education, covering the risks of diabetes for both the mother and the fetus, the importance of self-monitoring, and nutrition education. This will include introducing food groups, emphasizing the significance of meal control, providing information on food labels, and facilitating discussions on pregnancy-related concerns and coping strategies.
- Session 2: The second session will continue with diabetes education, focusing on the importance of exercise. Additionally, there will be discussions on pregnancy-related topics such as pain relief during labor and addressing issues of intimate partner violence.
- Session 3: The third session will address postpartum diabetes and what to expect during the postpartum period. Participants will also receive information on the importance of glucose tolerance testing. Pregnancy-related topics will include discussions on expectations during labor and delivery, as well as contraception.
  Session 4: The fourth session will emphasize diabetes education related to lifelong health monitoring and the risk of diabetes for offspring. Additionally, there will be discussions on pregnancy-related topics such as newborn care, including sleeping, proper usage of car seats, and feeding (Table 1).

**Table 1:** Curriculum for group prenatal care in women with diabetes

Sessions	Pregnancy Titles	Diabetes Titles
1	Introduction, discussion about expectations from the group, measurement of weight and blood pressure, concerns and normal pregnancy disorders, and coping strategies	Explanation of diabetes risks for mother and fetus and the importance of self-monitoring/introduction of food groups and the importance of meal control/introduction of food labels
2	Introduction, discussion about expectations from the group, measurement of weight and blood pressure, methods of pain relief during childbirth and intimate partner violence	Exercise
3	Introduction, discussion about expectations from the group, measurement of weight and blood pressure, what to expect during labor and delivery, prevention of pregnancy and breastfeeding	Diabetes after pregnancy and postpartum issues, and the importance of glucose tolerance test
4	Introduction, discussion about expectations from the group, measurement of weight and blood pressure, infant care (sleep/car seat/feeding)	Lifelong health monitoring and the risk of developing diabetes for children

The control group will receive standard care based on national guidelines.

#### Outcomes

The primary outcomes of the quantitative phase will include the mean scores of diabetes self-care activities, diabetes management self-efficacy, and pregnancy experience. The secondary outcomes will involve comparing the mean scores of satisfaction with antenatal care, rate of preterm birth, rate of cesarean section, neonatal hospitalization in the neonatal intensive care unit, breastfeeding performance score at 6 weeks postpartum, and validation of the SDSCA tool.

#### Data collection

##### Socio-demographic questionnaire

This questionnaire includes questions about age, education level, occupation, marital status, living situation, family income sufficiency, type of diabetes, family history of diabetes, and etc.

##### Obstetric history questionnaire

This questionnaire includes questions about the number of pregnancies and childbirths, previous mode of delivery, type of diabetes, previous history of gestational diabetes, preference for infant gender, unintended pregnancy, experience of violence during pregnancy, need for medication treatment, and duration of type 2 diabetes.

##### Maternal and neonatal outcomes checklist

This questionnaire includes questions about preterm birth, cesarean section, neonatal hospitalization in the neonatal intensive care unit.

##### The diabetes management self-efficacy scale

The Diabetes Management Self-Efficacy Scale (DMSES) is a tool used to measure perceived self-efficacy in managing type 2 diabetes. It consists of a 5-point Likert scale with 20 items and four subscales related to nutrition, weight control, medical care, and blood glucose monitoring. The total score ranges from 0 to 200, with higher scores indicating higher self-efficacy for healthy behaviors. The scale has demonstrated good reliability with a Cronbach’s alpha of 0.81 and test-retest reliability of 0.79 [27, 28]. In a recent study, the Arabic version of the scale was validated for women with gestational diabetes, showing good content validity and a Cronbach’s alpha of 0.85 [29].

##### Pregnancy experience scale

The Pregnancy Experience Scale (PES) is a 20-item questionnaire that assesses two domains, “Uplifts” and “Hassles,” during pregnancy. It includes ten items for measuring hassles and ten items for measuring uplifts. Participants rate each item on a four-point Likert scale, with higher scores indicating greater levels of hassles or uplifts experienced during pregnancy [30]. The English version of the PES questionnaire exhibits strong internal consistency, with an overall reliability coefficient of 0.80. Specifically, the “Hassles” domain has a reliability coefficient of 0.82, and the “Uplifts” domain has a reliability coefficient of 0.83. In Iran, the Persian version of the PES questionnaire demonstrates acceptable reliability, with an overall reliability coefficient of 0.71. The “Hassles” domain has a reliability coefficient of 0.77, while the “Uplifts” domain shows good consistency with a reliability coefficient of 0.67 [31].

##### Visual Analog Scale (VAS)

Measuring the level of satisfaction with antenatal care using the Visual Analog Scale (VAS) involves a graded line ruler that is 10 centimeters long. The woman is required to indicate her level of satisfaction on this graded line, with zero representing dissatisfaction and 10 representing the highest level of satisfaction [32].

##### Breastfeeding performance

Breastfeeding performance will be assessed in this study using a questionnaire developed by Agunbiade and Ogunleye, 6 weeks after childbirth. The questionnaire consists of six items, and scoring 4 or higher on the items indicates good breastfeeding performance, based on national guidelines [33].

##### Summary of the diabetes self-care activities questionnaire

The SDSCA is an assessment tool used to measure various aspects of diabetes management. It consists of 11 items that cover areas such as diet, exercise, glucose monitoring, foot care, and smoking. Responses are rated on a scale from 0 to 7 days per week [34]. The tool has shown good internal consistency with high inter-item correlations (0.47) and moderate test-retest reliability (0.40). However, for pregnant women with diabetes, only three areas (diet, exercise, and glucose monitoring) are evaluated because foot care and smoking are not relevant in this context [35].

#### Validation of the SDSCA questionnaire

The psychometric validation of the SDSCA questionnaire on pregnant women with diabetes in Iran has not been conducted based on the available research. At the outset of the study, written consent will be obtained from the creator of the questionnaire. To establish the validity of the questionnaires, various methods such as translation validity (Forward & Backward Translation), content validity, face validity, and construct validity will be employed.

During the process of translating the questionnaires, the questionnaire items will first be semantically translated from English to Farsi to ensure the preservation of the original version’s concepts and meanings. The initial translations will then undergo review and revision by another translator or a team of reviewers. Subsequently, the merged version will be translated back into the original language, and the final translation will be carried out by a team of translators proficient in both languages. Finally, the translated questionnaire will be reviewed by three to four translators to ensure the accuracy and correctness of the linguistic, semantic, and conceptual aspects of the translation. This meticulous process is implemented to guarantee the precision and accuracy of the questionnaire translation across languages [36].

The face validity of the SDSCA questionnaire will be evaluated using a combination of qualitative and quantitative methods. The qualitative approach aims to identify any ambiguities, inadequacies, or difficulties in understanding the questionnaire items. It also ensures that the items are relevant and appropriate for the intended purpose. Furthermore, the quantitative method known as the impact score will be utilized, with scores above 1.5 considered indicative of acceptable face validity [37].

To assess content validity, a qualitative method will be employed to examine grammar, vocabulary, item importance, placement, and completion time of the questionnaire. Additionally, the quantitative approach involves calculating the Content Validity Ratio (CVR) and the Content Validity Index (CVI) to determine the adequacy and representativeness of the questionnaire’s content.

The construct validity of the tool will be assessed through exploratory factor analysis (EFA) and confirmatory factor analysis (CFA). EFA will help identify underlying factors or dimensions in the data, while CFA will validate the hypothesized factor structure.

The reliability of the tool will be evaluated using the test-retest method, which assesses reproducibility over time. The intra-class correlation (ICC) coefficient will be calculated to determine agreement between repeated administrations. Internal consistency will also be assessed using Cronbach’s alpha coefficient, which measures the coherence and reliability of the tool’s items [38].

##### Sample size required for scale psychometrics

Considering that Nunnally and Bernstein recommend a minimum of 5 samples per item for factor analysis [39], in this study, the sample size based on the 11 items in the SDSCA questionnaire is 55. However, considering that EFA and CFA will be conducted on two separate data sets, 110 participants will be selected.

### Data management

The collected data will be entered into SPSS software version 24 promptly after collection to ensure accuracy. The entries from selected participants will undergo a thorough review to further enhance data accuracy. Regular reminders will be implemented to promote participant adherence and engagement in the study. Informed consent will be obtained, allowing for the use of both electronic and paper records to recover any potentially lost data.

### Management of missing data

To minimize missing data, interviews will be conducted by the research team to complete questionnaires and checklists. Strategies including informed consent, comprehensive follow-up data collection, standardized checklists, and frequent follow-ups will reduce loss to follow-up. In cases of non-adherence or withdrawal, participant outcomes will still be investigated and reported. Missing data will be addressed using multiple imputation, and sensitivity analysis will compare results of modified intention-to-treat analysis with imputed data analysis.

### Confidentiality

To maintain complete confidentiality, participant identification data will not be included in questionnaires or computer software. Unique codes are used for participant identification. Identifiable information is recorded separately and securely stored. Access is limited to authorized individuals, with exceptions granted for valid justifications.

### Data analysis

The data obtained will be analyzed using the double data entry approach in SPSS software version 24. Descriptive statistics, including frequency (percentage) for categorical data and mean (standard deviation) for normally distributed data, as well as median (interquartile range) for non-normally distributed data, will be used to describe socio-demographic and obstetrics characteristics. The normality of the data will be assessed using the Kolmogorov-Smirnov test.

In the bivariate analysis, ANCOVA and the chi-square test will be employed to compare the means of variables related to diabetes self-care activities, diabetes management self-efficacy, pregnancy experience, satisfaction with perinatal care, breastfeeding performance at 6 weeks postpartum, and the frequencies of preterm delivery, cesarean section delivery, and neonatal hospitalization between the two groups. In the multivariate analysis, the general linear model will be utilized, controlling for socio-demographic and obstetric characteristics, to further explore the relationships among the variables mentioned above.

### Qualitative phase (phase 2)

#### Study design

In the qualitative phase of this study, the research method utilized is qualitative content analysis with a conventional approach [40].

#### Specific objectives (qualitative phase)

The specific objectives of the second phase are to explore the understanding of pregnant women with diabetes regarding the impact of implementing the Centering Pregnancy group care model on their care process.

#### Eligibility criteria

Participants who are part of the Centering Pregnancy group care model and express willingness to participate in the study.

#### Sample size and sampling

The selection of participants will be based on the objective of the study, which takes place after the completion of training sessions. Maximum diversity will be taken into consideration regarding individual, social, and maternal characteristics. The sampling process will continue until information saturation is achieved, which means that no new information or codes will be obtained.

#### Data collection

As part of the qualitative research methodology, in-depth and semi-structured individual interviews will be conducted. Open-ended questions will be utilized during these interviews to allow for rich and detailed responses from participants. The interviews will take place in natural settings, provided suitable conditions exist, and participants express their willingness to participate.

Prior to this phase, the research team will collaborate to develop the interview guide, ensuring the collection of valid data and alignment with the research objectives. The interviews will commence with predetermined questions, and as the dialogue progresses, probing and exploratory questions will be employed to gain a more profound understanding of the participants’ experiences [41]. To ensure accurate documentation, the researcher will record non-verbal cues, such as vocal intonation, facial expressions, and body language, on a dedicated record sheet, noting the time and location of each interview.

#### Data analysis

The qualitative content analysis method will be used for data analysis in this study [42], involving categorization, coding, and identification of themes and patterns in the textual data. This analysis aims to uncover hidden themes and patterns in the participants’ data. Categories will be created to group content with common characteristics, and further subcategories may be formed. Themes will capture the “how” aspect of the data, representing meaningful threads within the coded data or categories at different interpretive levels. The interview notes will be transformed into categories and themes as part of the analysis process. The organization of interview texts and codes will be facilitated through the utilization of MAXQDA software.

To ensure the quality of the qualitative research, five criteria are commonly used: credibility or acceptability, dependability, confirmability, transferability, and authenticity. These criteria help evaluate the accuracy and trustworthiness of the research findings [43, 44].

### Combination of both quantitative and qualitative phase (phase 3)

#### Specific objectives

The specific combined objective is to provide a better understanding of the impact of implementing the Centering Pregnancy group care model on maternal and infant outcomes by elucidating women’s perception of the model’s influence on their care process.

#### Data integration

Data integration can be achieved through concurrent designs, including concurrent triangulation, concurrent nested, and concurrent transformative. In concurrent triangulation, both qualitative and quantitative data are collected and analyzed simultaneously, with equal priority given to both types of data. Data analysis is typically conducted separately, and integration occurs during the interpretation phase [45]. The current research involves collecting numerical data for the quantitative phase and textual data for the qualitative phase. The collected data will be analyzed separately using SPSS and content analysis, respectively. The findings from both phases will be integrated, and a comprehensive interpretation will be presented.

#### Ethics approval and consent to participate

The present study followed ethical guidelines, including the Helsinki Declaration and national principles. Approval was obtained from the ethics committee of Tabriz University of Medical Sciences (IR.TBZMED.REC.1402.652/ 2023-12-04). The quantitative phase, a randomized controlled clinical trial, is registered in the Iranian Clinical Trial Registration Centre (IRCT20120718010324N80/ 2024-01-03). Informed written consent will be obtained from all participants, ensuring confidentiality and privacy. Participants have the right to withdraw from the study at any stage without consequences, and non-cooperation is voluntary without affecting their services.

## Discussion

The WHO recommends the provision of health system interventions to improve the use and quality of antenatal care in order to enhance perinatal outcomes and women’s experience of care [46].

Antenatal care can be delivered through individual or group-based approaches [47]. In low-risk pregnant women, group antenatal care has been shown to yield improvements in stress levels, self-confidence, knowledge, motivation for healthy behaviors during pregnancy, and active engagement in healthcare practices [48].

Women diagnosed with diabetes during pregnancy often experience shock and anxiety. They may also feel guilt regarding the potential effects on their unborn child. These feelings of anxiety and remorse can have a negative impact on their overall pregnancy experience [49, 50]. It has been shown that group-based antenatal care has a positive impact on the psychosocial well-being of women with higher levels of stress or lower personal coping resources [51].

Group-based antenatal care, specifically the Centering Pregnancy model, has been associated with higher utilization of long-acting reversible contraception and increased likelihood of postpartum oral glucose tolerance testing in women with diabetes. However, no significant differences in adverse outcomes were found between group-based and individual care, warranting further research [19].

Lifestyle interventions can enhance women’s self-management of diabetes and reduce stress and anxiety during pregnancy [52, 53]. Education regarding GDM control, healthy eating, and physical activity are practical interventions that improve self-care and empower women with diabetes [54, 55]. Centering Pregnancy group prenatal care model, has demonstrated encouraging outcomes in improving nutrition and lifestyle among women with diabetes [32]. However, existing studies on the Centering Pregnancy model have predominantly focused on healthy pregnant women, and there is a lack of qualitative or mixed-methods research specifically investigating its effectiveness in pregnant women with diabetes [48, 56–58]. Nevertheless, it is recommended to consider implementing this care model for high-risk women to enhance a comprehensive understanding and achieve better outcomes [14, 59].

### Strengths and limitations

The present study will be the first of its kind in Iran to implement and evaluate the Centering Pregnancy group care model in pregnant women with diabetes using a mixed methods approach. Other strengths include the use of randomization methods to prevent selection bias in the quantitative phase and the validation of the SDSCA questionnaire, which measures self-care activities, in Iranian women with GDM and pregnant women with type 2 diabetes for the first time. This questionnaire can serve as a standardized and widely used tool for assessing self-care activities in these women in future studies. Furthermore, exploring the experiential understanding of women with diabetes in pregnancy regarding this group care model can provide healthcare professionals with greater knowledge about women’s experiences with diabetes and inform practical interventions for reducing the risk of diabetes in women.

The study has several limitations. Firstly, the reliance on self-report measures, such as the SDSCA questionnaire, may introduce recall bias or social desirability bias. Additionally, there are constraints associated with financial limitations the ability to assess long-term outcomes. Another limitation is the lack of blinding among the providers and participants, which is inherent to the nature of the intervention and could potentially bias the results. However, the statistical analyst will be unaware of the group assignments during the analysis, minimizing this bias. Furthermore, since this type of care is being implemented for the first time in Iran, it relies on the cooperation and acceptance of women, which could lead to attrition bias in the 8-week intervention. Lastly, the generalizability of the study’s findings is limited to women with gestational diabetes and low-risk overt diabetes.

## Conclusions

By evaluating the effectiveness of the Centering Pregnancy group care model in improving antenatal services and integrating quantitative and qualitative findings, this study aims to contribute to the enhancement of care, the promotion of positive experiences, and the improvement of self-efficacy among women with gestational diabetes mellitus (GDM) and low-risk type 2 diabetes. As a result, the implementation of this group care approach is expected to empower women in effectively self-managing their condition, thereby leading to improved maternal and neonatal outcomes. Additionally, the adoption of such an approach has the potential to reduce the financial burden of diabetes on the healthcare system in Iran.

## Data Availability

No datasets were generated or analysed during the current study.

## Abbreviations

GDM: Gestational diabetes mellitus
NICU: Neonatal intensive care unit
SDSCA: The Summary of Diabetes Self-Care Activities
WHO: World Health Organization
